# IoMT–Fog–Cloud-based AI frameworks for chronic disease diagnosis: updated comparative analysis with recent AI-IoMT models (2020–2025)

**DOI:** 10.3389/fmedt.2026.1748964

**Published:** 2026-01-22

**Authors:** Kitsakorn Locharoenrat

**Affiliations:** Department of Physics, School of Science, King Mongkut’s Institute of Technology Ladkrabang, Bangkok, Thailand

**Keywords:** chronic disease diagnosis, cloud computing, deep learning, fog computing, internet of Medical Things (IoMT), network performance

## Abstract

Chronic diseases such as diabetes and cardiovascular disease require frequent monitoring and timely clinical feedback to prevent complications. Internet of Medical Things (IoMT) systems increasingly combine near-patient sensing with Fog and Cloud computing so that time-critical preprocessing and inference can run close to the patient while compute-intensive training and population-level analytics remain in the Cloud. This review synthesizes primary studies published between 2020 and 2025 that implement AI-enabled IoMT, with an emphasis on systems that report both diagnostic performance and network quality-of-service (QoS). Following PRISMA 2020, we screened database records and included 14 primary studies; we focus the joint performance–QoS synthesis on six IoMT–Fog–Cloud frameworks for diabetes and cardiovascular disease and compare them with two recent multi-disease AI-IoMT models (DACL and TasLA). Diabetes-oriented implementations commonly report accuracy around 95%–96% using explainable or ensemble deep learning, whereas some cardiovascular frameworks report >99% accuracy in controlled settings; we therefore discuss plausible sources of optimistic performance, including small datasets, class imbalance, curated benchmarks, and potential leakage/overfitting in simulation-based evaluations. Across IoMT–Fog–Cloud studies, placing preprocessing and/or inference at the Fog layer repeatedly reduces end-to-end latency for streaming biosignals, but multi-Fog provisioning can increase energy and power demands. To support more reproducible comparisons, we organize 14 extracted metrics into (i) diagnostic performance (accuracy, precision, recall, F1-score, sensitivity, specificity) and (ii) system/network QoS (latency, jitter, throughput, bandwidth utilization, processing/execution time, network usage, energy consumption, power consumption), and we translate the evidence into study-linked design recommendations for future deployments.

## 1 Introduction

Chronic non-communicable diseases—including type 2 diabetes mellitus, gestational diabetes, cardiovascular disease, hypertension, and obesity—account for a large share of global morbidity and mortality. Managing these conditions requires frequent monitoring of physiological parameters and timely feedback to patients and clinicians, which is difficult to achieve with traditional hospital-centric care models.

The Internet of Medical Things (IoMT) addresses this challenge by connecting wearable sensors, home monitoring devices, and medical equipment to communication networks and Cloud services. By combining IoMT with Fog and Cloud computing, data can be preprocessed and analyzed near the data source (Fog nodes) while computationally intensive deep learning models run in the Cloud (1–3). This IoMT–Fog–Cloud paradigm offers a flexible way to balance latency, energy consumption, bandwidth, and security while maintaining high diagnostic accuracy for chronic disease diagnosis and prognosis (4–6, 12–14).

Recent research has produced multiple IoMT–Fog–Cloud frameworks tailored to specific diseases, including diabetes mellitus and cardiovascular disease (1–6). In parallel, new AI-IoMT architectures such as the Deep Auto-Optimized Collaborative Learning (DACL) model and the TasLA attention-based data fusion model have been proposed to support multi-disease prognosis in IoMT settings (7, 8). In addition, optimization-focused work and IoMT-enabled arrhythmia classification provide complementary advances in model design and hyperparameter tuning for medical AI systems (9–11). Separate efforts on chronic disease IoMT pipelines and fog-enabled health monitoring further expand the ecosystem of smart healthcare architectures (12–14).

These strands are often reported with different emphases: disease-specific IoMT–Fog–Cloud frameworks typically provide both diagnostic outcomes and at least some network/QoS measurements, whereas recent AI-IoMT models focus on classification accuracy and robustness with limited deployment-level evaluation. This review integrates both perspectives. We summarize six IoMT–Fog–Cloud frameworks for diabetes and cardiovascular disease (1–6), describing their sensing setups, Fog/Cloud roles, learning models, diagnostic performance, and reported network behavior. We then contrast these implementations with two recent multi-disease AI-IoMT models (DACL and TasLA) (7, 8) and draw on related IoMT and optimization studies to interpret design choices and practical constraints (9–14).

Recent surveys and systematic reviews have discussed fog/edge/cloud computing for smart healthcare and IoMT (e.g., emphasizing performance parameters such as latency, bandwidth, and scalability) (17, 18), mobile edge computing and QoS in IoMT systems (19), and QoS monitoring/optimization approaches for smart healthcare applications (20), as well as broader IoMT technology landscapes that touch on fog/edge layers (21). However, these prior reviews typically either (1) address architectures at a high level without extracting and harmonizing diagnostic and network/QoS outcomes from chronic-disease implementations, or (2) focus on QoS metrics without jointly comparing diagnostic performance, datasets, and validation strategies. In contrast, this review (Table 1) provides a PRISMA-guided synthesis of 14 key diagnostic and network/QoS metrics across six IoMT–Fog–Cloud chronic-disease frameworks and two recent multi-disease AI-IoMT models (2020–2025), and translates the evidence into actionable, study-linked design recommendations for system designers (Table 2). Specifically, the 14 metrics are: diagnostic (accuracy, precision, recall, F1-score, sensitivity, specificity) and network/QoS (latency, jitter, throughput, bandwidth utilization, processing/execution time, network usage, energy consumption, power consumption).

**Table 1:** Comparison with recent related reviews (2022–2024) and positioning of this work.

Review (year)	Review type	Architecture focus	Clinical scope	Metrics focus	How this work differs/adds
Kashyap et al. (2022) (17)	Survey	Fog + IoT/IoMT healthcare	Multiple conditions (broad)	Discusses performance parameters (e.g., latency, bandwidth) and challenges	No joint extraction/harmonisation of diagnostic + QoS outcomes for chronic-disease frameworks; not focused on trade-off analysis.
Navakauskas & Kazlauskas (2023) (18)	Systematic review	Fog computing in healthcare	Broad healthcare use cases	Trends/challenges; limited metric-level synthesis of diagnostic models	Does not perform a metric-by-metric trade-off synthesis across chronic-disease implementations.
Awad et al. (2023) (19)	Survey	Mobile edge computing (MEC) in IoMT + cloud integration	Broad IoMT applications	QoS/real-time considerations (e.g., low latency)	Focuses on MEC paradigm rather than Fog–Cloud chronic-disease diagnostic frameworks and does not extract diagnostic outcomes across datasets.
Younas et al. (2023) (20)	Systematic literature review	IoT edge devices for smart healthcare	Broad smart healthcare	QoS parameters (throughput, bandwidth, jitter, latency, etc.)	QoS-centric; does not jointly analyse diagnostic performance metrics alongside QoS for chronic-disease AI models.
Alturki et al. (2024) (21)	Review	IoMT standards/protocols; distinguishes fog vs. edge	Broad IoMT landscape	High-level challenges and research directions	Technology landscape review; does not perform PRISMA-guided metric extraction and cross-study comparison of diagnostic and QoS metrics.
This review (2020–2025)	PRISMA-guided systematic review	IoMT–Fog–Cloud for chronic-disease diagnosis + multi-disease AI-IoMT models	Diabetes + cardiovascular (+ multi-disease models)	Joint analysis of 14 metrics: 6 diagnostics + 8 QoS/system metrics	Provides harmonised metric extraction, cross-study synthesis of architecture–QoS patterns, and evidence-linked design recommendations (Table 2).

**Table 2:** Evidence-linked design recommendations derived from included studies.

Design consideration	Recommendation	Evidence (Ref.)	Metrics affected	Implementation notes
Fog vs. cloud task allocation	Place latency-critical preprocessing/inference at Fog; keep long-term storage/training in Cloud.	(4–6)	Latency, jitter, processing time	Use streaming pipelines for ECG/arrhythmia; ensure failover when Fog connectivity degrades.
Multi-fog scaling	Distribute workload across multiple Fog nodes when response-time constraints dominate, but budget for higher energy/power.	(1–3, 5)	Latency ↓; Energy/Power ↑ (often)	Profile communication overhead; consider adaptive offloading under load.
Bandwidth-aware data handling	Reduce upstream traffic via feature extraction/compression near IoMT/Fog before Cloud upload.	(1–6)	Bandwidth utilisation, network usage	Define sampling rate and windowing; report compression ratios and resulting accuracy impact.
Model optimisation for fog/edge	Adopt lightweight models and deployment-oriented optimisation (e.g., feature selection, attention, pruning/quantisation) for Fog inference.	(7, 8)	Processing time, energy; accuracy trade-offs	Validate on target hardware or realistic emulation; measure latency under representative network conditions.
Reporting standards	Report dataset provenance, class balance, validation strategy, and clearly define QoS measurement points/tools.	Across studies (Tables 3–5)	Reproducibility	Include both diagnostic and QoS metrics in the same experiment when possible; provide parameter settings for simulators and network conditions.
Evaluation realism	Complement simulations with prototype or real-world measurements where feasible.	Across studies	External validity	Report sensor type, link technology, and deployment setting (hospital/home/ambulance); discuss regulatory and clinical readiness.

## 2 Methods

### 2.1 Protocol and reporting standard

This study is reported in accordance with the PRISMA 2020 statement for systematic reviews. A review protocol was not registered; however, the eligibility criteria, search strategy, and data-extraction items were specified *a priori* to support reproducibility.

### 2.2 Information sources and search dates

We searched peer-reviewed literature published between 1 January 2020 and 31 May 2025 in IEEE Xplore, ScienceDirect (Elsevier), SpringerLink, MDPI, Hindawi/Wiley, PubMed, and Google Scholar. The final search was completed in May 2025. We also screened the reference lists of included articles to identify additional eligible studies within the same time window.

### 2.3 Search strategy

Search queries were constructed using controlled keywords and free-text terms and then adapted to the syntax of each database/publisher platform. The core Boolean expression was:

(“Internet of Medical Things” OR IoMT OR “medical IoT”) AND (fog OR edge OR “fog computing” OR “edge computing” OR cloud) AND (“chronic disease” OR diabetes OR “cardiovascular disease” OR “heart disease” OR arrhythmia OR stroke) AND (“machine learning” OR “deep learning” OR AI) AND (latency OR jitter OR throughput OR “quality of service” OR QoS OR energy OR power).

Search results were exported (where supported), de-duplicated, and then screened against the eligibility criteria.

### 2.4 Eligibility criteria

Inclusion criteria:

Primary research articles proposing an IoMT, Fog, Cloud, or hybrid IoMT–Fog–Cloud architecture for chronic disease diagnosis or prognosis.
Focus on at least one chronic disease (e.g., diabetes, cardiovascular disease/arrhythmia, stroke) and use of AI models (machine learning or deep learning).
Reporting at least one diagnostic performance metric (e.g., accuracy, precision/recall, F1-score, sensitivity, specificity).
For the IoMT–Fog–Cloud subgroup analysis, reporting at least one network/QoS metric (e.g., latency, jitter, throughput, bandwidth utilization, processing time, energy/power).

Exclusion criteria:

Non-English articles.
Studies outside the 2020–2025 time window.
Purely conceptual frameworks without experimental evaluation.
Studies that do not use machine learning/deep learning for the diagnostic task.

### 2.5 Study selection and PRISMA flow

**Figure 1:**
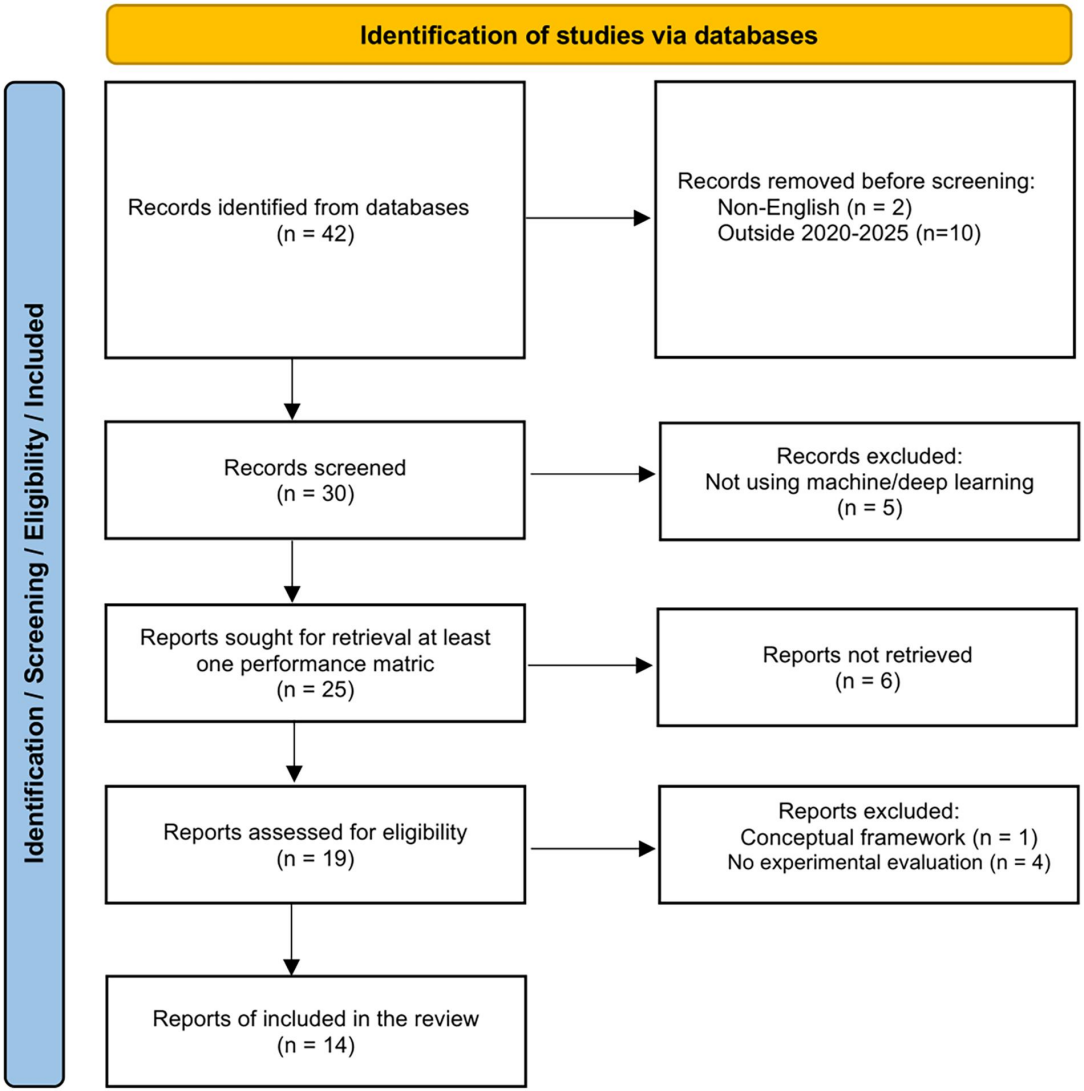
PRISMA 2020 flow diagram for study selection (databases only).

Figure 1 summarizes the selection process following PRISMA 2020. The search identified 42 records. After removing 12 records before screening (2 non-English and 10 outside 2020–2025), 30 records were screened. Five records were excluded because they did not use machine/deep learning. Twenty-five reports were sought for retrieval; six could not be retrieved. Nineteen reports were assessed for eligibility, and five were excluded (one conceptual framework and four without experimental evaluation). Fourteen studies were included in the final review.

### 2.6 Rationale for focusing on six IoMT–Fog–Cloud frameworks and two AI-IoMT models

Fourteen primary studies met the eligibility criteria. Of these, six implemented an explicit three-tier IoMT–Fog–Cloud (or close equivalent) architecture and reported at least one network/QoS metric; these six form the core set for the joint performance–QoS synthesis (Tables 3,4). To reflect recent algorithmic developments in multi-disease AI-IoMT, we also included two representative models—DACL and TasLA—that report strong diagnostic performance but provide limited network/QoS evaluation. Accordingly, they are compared mainly on AI design choices and diagnostic metrics (Table 5) and discussed in terms of likely deployment constraints. The six IoMT–Fog–Cloud frameworks were prioritized because they (1) instantiate a three-tier architecture, (2) report both diagnostic and network/QoS metrics (enabling trade-off analysis), and (3) provide architectural diversity (sensing modalities, Fog task allocation, and evaluation environments) with sufficient reporting detail for metric extraction. When several eligible studies addressed the same disease task, we favored papers with more complete metric reporting and higher citation impact. DACL and TasLA were selected because they introduce attention/fusion and optimization strategies that are increasingly used in medical AI and are plausible candidates for future IoMT deployments, even when QoS metrics are not yet routinely reported.

**Table 3:** IoMT–Fog–Cloud frameworks for diabetes and cardiovascular disease.

Study	Ref. no.	Disease type	Sample size	Number of features	Dataset source (public/clinical/hybrid)	Validation strategy (as reported)	Deployment realism (simulation vs. real-world)	Main AI model(s)	Architecture (IoMT/Fog/Cloud)	Accuracy (%)	F1-score (%)	Network metrics/notes
Pati et al., 2022	(1)	Type 2 diabetes mellitus	2,768	8	Pima Indians Diabetes (UCI) + Hospital Frankfurt Diabetes (Kaggle)	NR	Simulation/scenario-based	ANN with bagging and voting ensembles	IoT sensors → Fog ANN → Cloud DNN baseline	≈94.5	≈95.0	Latency and energy depend on Fog-node count; multi-Fog reduces latency but increases power consumption.
El-Rashidy et al., 2023	(2)	Gestational diabetes	16,354	21	MIMIC-III EHR	NR	NR (retracted; interpret cautiously)	Explainable deep neural network (DNN)	IoMT devices → Fog for preprocessing → Cloud DNN	≈95.7	≈93.7	End-to-end latency reported at millisecond scale in Fog–Cloud deployment (retracted 21 Nov 2024; interpret cautiously).
Yıldırım et al., 2023	(3)	Type 2 diabetes mellitus	200	5	East Jakarta clinics + public datasets	NR	Simulation/scenario-based	Fuzzy logic + ML classifiers (e.g., SVM)	IoMT sensors → Fog processing → Cloud storage and analytics	≈89.5	≈90.1	Reports latency, jitter, and energy for multiple network scenarios; moderate latency with constrained resources.
Sun et al., 2020 (FogMed)	(4)	Atrial fibrillation/arrhythmia	84	4 ECG-derived features	Long-term ECG recordings	NR	Prototype/near-real-time	Stacked LSTM (FogMed framework)	Wearable ECG → Fog LSTM → Cloud storage	≈92.0	≈92.0	Demonstrates feasibility of real-time AF detection at the Fog layer.
Pati et al., 2023	(5)	Cardiovascular disease	920	10	Heart-disease datasets (UCI repository)	NR	Simulation/scenario-based	DNN with bagging and voting	IoT sensors → Fog DNN → Cloud comparison models	≈94.3	≈96.8	Latency, jitter, throughput, and energy evaluated for master-only, master + Fog, and Cloud-only setups.
Nancy et al., 2023	(6)	Cardiovascular disease	≈597–100,000	12	Hybrid of real and synthetic heart-disease datasets	NR	Simulation/scenario-based	GNU-RNN (gated recurrent unit-based RNN)	IoMT devices → Fog GNU-RNN → Cloud analytics	≈99.1	≈99.1	Very low latency reported for Fog deployment; near-perfect specificity and accuracy.

**Table 4:** Definitions and measurement context for network QoS metrics across included IoMT–Fog–cloud studies.

Study (Ref.)	QoS metric(s) reported	Definition/measurement point (as reported)	Evaluation environment	Tool/notes
Pati et al., (1)	Latency; network use; arbitration time; energy/power	NR (reported as system/network-level metrics under varying Fog-node counts)	Simulation/scenario-based	NR
El-Rashidy et al., (2)	End-to-end latency	NR (reported as end-to-end latency in Fog–Cloud deployment)	NR	Retracted (21 Nov 2024); interpret cautiously.
Yıldırım et al., (3)	Latency; jitter; processing time; energy/power	NR (reported across multiple network configurations)	Simulation/scenario-based	NR
Sun et al., (FogMed) (4)	Real-time responsiveness (latency NR)	NR (focus on Fog-layer real-time AF detection feasibility)	Prototype/near-real-time	NR
Pati et al., (5)	Latency; jitter; throughput; energy/power	NR (reported for master-only vs. master + Fog vs. Cloud-only scenarios)	Simulation/scenario-based	NR
Angel Nancy et al., (6)	Latency (very low); energy/power (*N*R)	NR (latency reported for Fog deployment; definition not standardised)	Simulation/scenario-based (inferred from scenario-based evaluation)	NR

**Table 5:** Multi-disease AI-IoMT frameworks.

Study	Ref. no.	Diseases covered	Dataset(s) (as reported)	Validation strategy (as reported)	Deployment realism (simulation vs. real-world)	IoMT architecture	Core AI components	Optimisation strategy	Main reported benefits	Network considerations
Nandagopal et al., 2024 (DACL)	(7)	Diabetes, heart disease, stroke	NR	NR	Algorithmic (dataset-based); network/QoS not evaluated	Three-tier AI-IoMT: sensors → wireless device communication → AI computing layer	Deep auto-encoder model (DAEM), Collaborative bias integrated GAN (ColBGaN)	Golden flower optimisation (feature selection), water drop optimisation (loss tuning)	Improved accuracy and reduced error compared with baseline ML/DL models on multiple datasets; lower computational cost.	Focuses on AI architecture; network metrics (latency, bandwidth) not explicitly quantified.
Khadidos et al., 2023 (TasLA)	(8)	Multiple chronic and acute conditions on benchmark datasets	NR	NR	Algorithmic (dataset-based); network/QoS not evaluated	IoMT sensors and gateways linked to Cloud analytics	Attention-based deep convolution network for data fusion	Tasmanian Devil-inspired optimisation and Lichtenberg optimisation for feature weighting	Higher classification accuracy and fewer false predictions than competing deep learning models; robust data fusion from heterogeneous sources.	Emphasis on algorithmic performance; IoMT network behaviour and QoS parameters are not reported in detail.
Selvarajan et al., 2023	(9)	Excretory-organism classification	NR	NR	Algorithmic (dataset-based); network/QoS not evaluated	AI-IoMT setting for medical image recognition	Artificial neural networks (ANNs)	Comparative evaluation across optimisation and training strategies	Demonstrates strong recognition performance on excretory-organism datasets.	Network aspects not the primary focus.
Kumar et al., 2023	(10)	Cardiac arrhythmia	NR	NR	Algorithmic (dataset-based); network/QoS not evaluated	IoT-enabled ECG acquisition	Deep model trained with enhanced hunt optimisation	Metaheuristic optimisation of model parameters	Improved arrhythmia classification accuracy over baseline methods.	Network performance (latency, jitter) not explicitly quantified.
Selvarajan, 2024	(11)	General engineering optimisation	NR	NR	Algorithmic (dataset-based); network/QoS not evaluated	Not specific to IoMT but relevant for AI-IoMT optimisation	Survey and analysis of modern optimisation techniques	Comparative study of multiple optimisation families	Provides guidance for choosing optimisation algorithms in AI-IoMT frameworks.	Not an IoMT system; used as methodological background.
Alugonda & Kodati, 2025 (EDCNN)	(12)	Heart disease	NR	NR	Algorithmic (dataset-based); network/QoS not evaluated	IoMT + cloud-based healthcare	Enhanced deep learning-assisted CNN (EDCNN)	ML-based hyperparameter optimisation for CNN	High accuracy heart-disease prediction using IoMT data.	Focus on predictive performance; network behaviour discussed qualitatively.

### 2.7 Data extraction, data items, and synthesis

From each included study we extracted (i) diagnostic performance outcomes and (ii) system/network QoS outcomes when reported. Extracted items included disease type, dataset source, sample size, number of features, AI model(s), architectural placement (IoMT/edge, Fog, Cloud), validation strategy (when described), and the reported metrics. Because the studies differ substantially in datasets, validation protocols, and network-evaluation methods, we synthesized results narratively rather than conducting a meta-analysis. For consistency, we defined 14 key metrics: six diagnostic metrics (accuracy, precision, recall, F1-score, sensitivity, specificity) and eight system/network metrics (latency, jitter, throughput, bandwidth utilization, processing/execution time, network usage, energy consumption, power consumption).

Performance index:

Accuracy, F1-score, precision, recall, sensitivity, and specificity.

Network/QoS index (for IoMT–Fog–Cloud systems):

Latency, jitter, throughput, bandwidth utilization, processing/execution time, network usage, energy consumption, and power consumption.

### 2.8 Evidence limitations and risk-of-bias considerations

To support cautious interpretation, we recorded potential sources of optimistic diagnostic performance during data extraction, including small or highly curated datasets, class imbalance, reliance on benchmark or synthetic data, unclear patient-level train–test separation, and possible leakage/overfitting—particularly in simulation-based Fog/Cloud evaluations. These considerations are discussed in Section 4.2.

## 3 Results

### 3.1 IoMT–Fog–Cloud frameworks for diabetes mellitus

The three diabetes-oriented frameworks target either type 2 diabetes mellitus or gestational diabetes prediction. Pati et al. (1) propose an IoMT–Fog–Cloud architecture in which wearable sensors and mobile devices send data to Fog nodes that perform pre-processing and deep learning-based classification, with additional benchmarking models deployed in the Cloud. Their ANN with bagging and voting ensembles achieves high accuracy and F1-score while exploring multiple network configurations (single master, master-plus-multi-Fog, and Cloud-only), revealing clear latency and energy trade-offs (1).

El-Rashidy et al. (2) propose a Fog–Cloud IoMT framework for gestational diabetes prediction using EHR data (MIMIC-III) and an explainable deep neural network. The paper reports strong performance (ACC ≈ 0.957) and millisecond-scale latency in the Fog–Cloud deployment; however, the article was later retracted (21 Nov 2024), so its quantitative results should be interpreted cautiously and are not used as primary evidence for conclusions.

Yıldırım et al. (3) propose a Fog–Cloud architecture for type 2 diabetes monitoring using data from East Jakarta health clinics and public repositories. Their framework relies on fuzzy logic and machine learning classifiers, achieving moderate accuracy but providing detailed measurements of processing time, latency, jitter, and energy consumption across different network configurations (3).

Table 3 summarizes the key characteristics of these three diabetes frameworks, including sample size, number of features, AI models, diagnostic performance, and network behavior (1–3).

### 3.2 IoMT–Fog–Cloud frameworks for cardiovascular disease

Sun et al. (4) introduce FogMed, a Fog-based framework for streaming ECG data and prognostic analysis. A stacked LSTM model operates at the Fog layer to detect atrial fibrillation from long-term recordings while the Cloud stores and aggregates data. This framework demonstrates that deep recurrent models can be deployed near the edge for real-time arrhythmia monitoring (4).

Pati et al. (5) extend their earlier diabetes architecture to cardiovascular disease. They use an IoMT–Fog–Cloud framework where Fog nodes host a deep neural network, combined with bagging and voting strategies, to classify coronary heart disease from multiple UCI datasets. The authors report strong accuracy and F1-score, while network simulations quantify latency, jitter, throughput, and power consumption under different Fog node counts (5).

Angel Nancy et al. (6) present a Fog-based smart cardiovascular disease prediction system that leverages a gated recurrent unit-based recurrent neural network (GNU-RNN). Their design explores large-scale synthetic and real datasets, achieving very high diagnostic performance (near-perfect accuracy and specificity) while maintaining low end-to-end latency when Fog nodes are used as intermediate processing hubs (6).

Table 3 also includes these cardiovascular systems to enable cross-disease comparison of sensor types, AI models, and network metrics (4–6).

### 3.3 Recent AI-IoMT frameworks for multi-disease prognosis

To extend the comparison beyond disease-specific IoMT–Fog–Cloud frameworks, we reviewed two recent multi-disease AI-IoMT architectures and several related optimization-oriented studies. The DACL framework proposed by Nandagopal et al. (7) uses a three-tier AI-IoMT design: wearable sensors collect patient data; a deep auto-encoder imputes and normalizes missing values; Golden Flower Optimization performs feature selection; and a Collaborative Bias Integrated GAN (ColBGaN) performs multi-disease classification (e.g., diabetes, heart disease, stroke). Across the datasets evaluated, DACL reports lower classification error and improved accuracy compared with baseline machine learning and deep learning models (7).

TasLA, introduced by Khadidos et al. (8), presents a Tasmanian and Lichtenberg optimized attention-based deep convolution data-fusion model for IoMT smart healthcare. Experiments on benchmark healthcare datasets show higher classification accuracy and fewer false predictions than competing deep learning models, highlighting the importance of optimization and attention mechanisms in multi-source IoMT data fusion (8).

Complementary work by Selvarajan et al. (9) on excretory-organism recognition and by Kumar et al. (10) on IoMT-enabled arrhythmia classification illustrates the use of deep learning and optimization for other medical pattern-recognition tasks. Selvarajan (11) surveys modern optimization techniques relevant to engineering and AI-IoMT systems. Nigar et al. (12) present an IoMT-to-Cloud chronic disease diagnosis pipeline, while Xhaferra et al. (13) and Suleiman et al. (14) report recent IoMT–Fog–Cloud models for diabetes and heart-disease prediction, respectively.

Table 5 summarizes the high-level architecture, disease coverage, optimization strategies, and main reported benefits of DACL and TasLA (7, 8).

### 3.4 Cross-study synthesis: architectural patterns and QoS reporting

Because QoS metrics were reported with inconsistent definitions, simulation tools, and measurement layers, direct numeric comparisons across studies should be treated cautiously. Table 4 therefore summarizes each metric's definition and measurement context (when available) to make differences in evaluation assumptions transparent.
Cardiovascular systems more frequently reported network/QoS metrics than diabetes-focused systems because many cardiovascular use cases (e.g., arrhythmia detection) are latency- and reliability-sensitive in near real-time monitoring, whereas glucose trend analysis is often less time-critical and therefore evaluated primarily via diagnostic metrics (1–6).
Architectures that distributed tasks across multiple Fog nodes (or used microservice-style decomposition) frequently reported lower latency/processing time at the expense of higher energy/power consumption due to additional Fog provisioning and inter-node communication (1–3, 5).
Multi-tier offloading strategies (Edge/IoMT → Fog → Cloud) tended to achieve lower response times than Cloud-only baselines, but the magnitude of gain depended strongly on workload partitioning, network topology, and the evaluation tool (simulation vs. prototype) (1–6).
Fog-layer placement of time-critical preprocessing and/or inference repeatedly reduced end-to-end latency for streaming biosignals (e.g., ECG/arrhythmia monitoring), while the Cloud was used for archival storage and computationally heavy analytics/training (4–6).

Overall, the included studies point to recurring architectural patterns that explain trade-offs between diagnostic performance and network/QoS behavior, and they help clarify why some disease domains report QoS metrics more consistently than others.

## 4 Discussion

### 4.1 Diagnostic performance across disease groups

Across diabetes-oriented IoMT–Fog–Cloud frameworks, reported diagnostic performance generally lies in the high-80s to mid-90s. Ensemble strategies and explainable deep models can improve accuracy and F1-score while maintaining low-latency inference at the Fog layer (1–3). Importantly, several studies rely on public benchmark datasets (e.g., UCI) or mixed public–clinical sources, which can simplify preprocessing and reduce data noise compared with real-world clinical streams.

Cardiovascular and arrhythmia frameworks often report higher peak performance. FogMed demonstrates the feasibility of real-time atrial fibrillation detection from continuous ECG streams using Fog-layer LSTM processing (4). Other heart-disease frameworks report very high (sometimes near-perfect) accuracy/precision/recall under optimized configurations (5, 6). These results highlight the promise of Fog-assisted inference for time-sensitive conditions, but they must be interpreted in the context of dataset characteristics and validation methodology (Section 4.2).

Recent multi-disease AI-IoMT frameworks such as DACL and TasLA extend beyond single-disease prediction by combining deep feature learning, meta-heuristic optimization, and data-fusion strategies across heterogeneous inputs (7, 8). However, these studies typically emphasise algorithmic performance on curated datasets and provide limited reporting of IoMT network behavior (latency, jitter, energy) under realistic deployment constraints (7–11).

### 4.2 Interpreting very high diagnostic performance and risk of bias

Several included studies report extremely high diagnostic metrics (>99% accuracy or near-perfect precision/recall) in specific experimental settings (6). While these values are reported as stated in the source publications, cross-study comparison of point metrics can be misleading when datasets, class balance, and validation protocols differ. Additionally, one gestational-diabetes framework in our initial extraction was later retracted (2), underscoring the importance of ongoing evidence appraisal when conducting technology reviews.

Potential sources of optimistic performance include: (1) small datasets or limited patient diversity, (2) substantial class imbalance, (3) reliance on benchmark or partially synthetic datasets, and (4) incomplete reporting of data-splitting procedures (e.g., patient-level separation) and hyperparameter tuning. In Fog/Cloud simulation studies, additional risks include unintended data leakage (feature normalisation or selection performed before splitting) and overfitting to a single network configuration.

To make these limitations more explicit, Tables 3,5 now report dataset source, validation strategy (as described by the original authors), and deployment realism (simulation vs. real-world). For future work, we recommend reporting patient-level splitting, nested cross-validation or external validation on independent cohorts, and calibration/uncertainty measures alongside accuracy and F1-score to better reflect clinical generalizability.

### 4.3 Network performance, architectural trade-offs, and metric comparability

The IoMT–Fog–Cloud frameworks provide a rich set of network-level results, but direct comparability is limited because latency, jitter, throughput, and energy are often defined and measured differently across studies. Some papers report end-to-end latency, others report processing time at a specific layer, and several omit the measurement point, workload, or simulation/testbed configuration.

Despite these limitations, a consistent architectural trend emerges: moving inference closer to the patient (edge/Fog) reduces end-to-end delay and bandwidth demand, but may increase energy/power consumption and system complexity, especially when multiple Fog nodes are deployed to improve responsiveness (1–6). By contrast, Cloud-only approaches can simplify maintenance and centralise analytics but typically increase network delay and may be unsuitable for time-critical events (e.g., arrhythmia alarms).

To clarify definitions and measurement context, Table 4 summarizes the QoS metrics reported in each IoMT–Fog–Cloud study and notes, where available, the measurement point and evaluation environment. Future studies should specify metric definitions (e.g., end-to-end vs. layer-specific), system layer, workload, and testbed/simulator details to support fair benchmarking.

### 4.4 Design implications

Fog-layer inference is particularly beneficial for time-sensitive conditions (e.g., arrhythmia detection) where latency and reliability are clinically critical (4–6).
For chronic-disease screening tasks with less stringent time constraints, hybrid Fog–Cloud approaches can balance responsiveness with resource efficiency by keeping lightweight preprocessing/inference near the edge and heavier analytics in the Cloud (1–3, 5).
AI-IoMT models that achieve strong accuracy on benchmark datasets (e.g., DACL, TasLA) should be co-designed with deployment constraints (compute, energy, privacy, connectivity) and evaluated under realistic IoMT network conditions before clinical translation (7, 8).
Unified reporting of both diagnostic and network/QoS metrics is essential to compare architectures fairly and to support reproducible engineering decisions (Tables 3–5).

To make the design guidance more actionable, Table 2 maps each recommendation to evidence extracted from the reviewed implementations (e.g., Fog/Cloud task allocations and architectural choices associated with lower latency or reduced energy in Tables 3–5). Table 2 also lists practical engineering options—such as lightweight model design and deployment-oriented optimization (feature selection, attention, and training strategies reported in DACL and TasLA 7, 8)—for Fog/Edge execution. Finally, Table 2 includes a minimal reporting checklist (dataset provenance, validation strategy, and QoS definitions/measurement context) intended to improve reproducibility and support more reliable cross-paper comparisons.

### 4.5 Clinical readiness, translational barriers, and emerging methods

Although the reviewed engineering frameworks demonstrate promising diagnostic and QoS performance, clinical readiness requires additional evidence beyond retrospective accuracy metrics. Key translational considerations include prospective validation in diverse patient cohorts, robustness to sensor noise and missing data, interpretability/traceability of model decisions, cybersecurity and privacy-by-design (e.g., encrypted transmission and secure model updates), and regulatory requirements for software as a medical device (SaMD) and connected devices.

Looking ahead, rapid advances in data integration and high-dimensional biomedical measurement may inform future IoMT analytics pipelines. For example, recent spatial multi-omic profiling and neuroinflammation mapping technologies highlight how multi-modal data fusion can uncover clinically relevant patterns at high resolution (15). Spatially resolved perturbation screens similarly illustrate scalable approaches to linking complex biological signals with outcomes (16). While these methods are not IoMT deployments, they reinforce the importance of rigorous, transparent data pipelines and may inspire future hybrid sensing frameworks that combine remote monitoring with complementary multi-modal clinical data sources.

## 5 Conclusions

This comparative review summarizes how IoMT–Fog–Cloud architectures and recent AI-IoMT models are being applied to chronic disease diagnosis, and where evidence is strong vs. still preliminary. Across diabetes and cardiovascular implementations, many systems report high diagnostic performance; however, cardiovascular studies more often evaluate end-to-end behavior (latency, jitter, throughput, and energy) under different network configurations, enabling explicit trade-off analysis.

Recent multi-disease AI-IoMT models such as DACL and TasLA illustrate the potential of deep representation learning, metaheuristic optimization, and data fusion for multi-condition prognosis from heterogeneous inputs. Related work on arrhythmia classification, optimization methods, and other IoMT applications further broadens the model-design space. In most cases, however, these studies provide limited deployment-level evaluation, so it remains unclear how such models would behave under realistic IoMT network conditions, constrained Fog/Edge resources, and patient-facing reliability requirements.

Future frameworks should therefore co-design the learning pipeline with the IoMT–Fog–Cloud infrastructure and report diagnostic and system/network outcomes together. Such integrated evaluation is essential for scalable and safe chronic disease management when moving from prototypes and simulations to clinical deployments.

## Data Availability

The original contributions presented in the study are included in the article/Supplementary Material, further inquiries can be directed to the corresponding author.
